# Experimental Pipeline (Expipe): A Lightweight Data Management Platform to Simplify the Steps From Experiment to Data Analysis

**DOI:** 10.3389/fninf.2020.00030

**Published:** 2020-07-24

**Authors:** Mikkel Elle Lepperød, Svenn-Arne Dragly, Alessio Paolo Buccino, Milad Hobbi Mobarhan, Anders Malthe-Sørenssen, Torkel Hafting, Marianne Fyhn

**Affiliations:** ^1^Center for Integrative Neuroplasticity, University of Oslo, Oslo, Norway; ^2^Institute of Basic Medical Sciences, University of Oslo, Oslo, Norway; ^3^Department of Physics, University of Oslo, Oslo, Norway; ^4^Department of Informatics, University of Oslo, Oslo, Norway; ^5^Department of Biosystems Science and Engineering, ETH, Zurich, Switzerland; ^6^Department of Biosciences, University of Oslo, Oslo, Norway

**Keywords:** data management, Python (programming language), open source software (OSS), analysis, data sharing, data base (DB)

## Abstract

As experimental neuroscience is moving toward more integrative approaches, with a variety of acquisition techniques covering multiple spatiotemporal scales, data management is becoming increasingly challenging for neuroscience laboratories. Often, datasets are too large to practically be stored on a laptop or a workstation. The ability to query metadata collections without retrieving complete datasets is therefore critical to efficiently perform new analyses and explore the data. At the same time, new experimental paradigms lead to constantly changing specifications for the metadata to be stored. Despite this, there is currently a serious lack of agile software tools for data management in neuroscience laboratories. To meet this need, we have developed Expipe, a lightweight data management framework that simplifies the steps from experiment to data analysis. Expipe provides the functionality to store and organize experimental data and metadata for easy retrieval in exploration and analysis throughout the experimental pipeline. It is flexible in terms of defining the metadata to store and aims to solve the storage and retrieval challenges of data/metadata due to ever changing experimental pipelines. Due to its simplicity and lightweight design, we envision Expipe as an easy-to-use data management solution for experimental laboratories, that can improve provenance, reproducibility, and sharing of scientific projects.

## 1. Introduction

Experimental neuroscience is increasingly moving toward an integrative understanding of phenomena by simultaneously collecting data with a wide range of techniques including behavioral tasks, electrophysiology, imaging and genetics. Datasets from these types of experiments span a wide range of spatial and temporal scales. Often, the experimental setup is not finalized or rigidly predefined before data acquisition begins. Results may thus require additional branches of experimentation or re-evaluation of the setup. For example, results may initiate additional behavioral studies, or combining electrophysiology with imaging data. Also, the majority of research today is carried out by research fellows employed on temporary contracts, imposing a challenge for both continuation of projects and data sharing. Put simply, projects usually organically grow and mature through the experimental timeline. Moreover, the need for multi-modal approaches in neuroscience makes data management ever more challenging, complicating data sharing and open collaboration.

In this paper we introduce a data management tool called Expipe (Experimental pipeline) which enables data management to simply evolve and mature organically together with experiments in a semi-structured fashion.

To improve reproducibility in neuroscience, several (larger) initiatives point toward tools that facilitate sharing of data and code (Crook et al., 2013; Denker and Grün, 2016; Zehl et al., 2016; Gleeson et al., 2017). Part of the data management challenge comes from the wide range of formats produced by different experimental paradigms. Moreover, with increased size of datasets, researchers are often unable to carry all their data around on their laptops or store them on workstations. The possibility to query a metadata collection without retrieving entire datasets is therefore becoming more important.

Data and metadata managing tools typically differ in the amount of *a priori* imposed structure. In a structured database, fields are typically required to be predefined and are best suited for use cases where it is possible to predict the types of data and metadata that will be stored. In unstructured databases, fields typically evolve while the database is used and updated. Being highly flexibile, these types of databases are easy to use, but can be difficult to share across users as their evolved structure might not be intuitive or well-documented. The current tools that exist for experimental databases, can typically be described by one of those two categories.

DataNet (HarkȩŻlak et al., 2014) is a data management method and architecture that defines repositories which can be accessed by any programming language through REST-based APIs. The goal of DataNet is to deliver a scalable solution that facilitates reproducibility and is capable of handling large data volumes. DataNet is designed to be run on top of a platform-as-a-service (PaaS) provider, such as CloudFoundry. While DataNet can be regarded as an advanced data management solution, its setup and usage is not specific to neuroscience and may require existing experience in data management solutions.

Another effort toward a lightweight data management software is dtool (Olsson and Hartley, 2019). Dtool was mainly designed for bioinformatics/genomics data and it provides a solution to package data and metadata together. Dtool implements a CLI and a simple Python API to create datasets, and metadata are provided by the user when a new dataset is generated. The dtool framework, however, does not enforce or suggest any organization of the dataset, leaving it to the user.

Another proposed solution to organize and store complex metadata is the odML (Grewe et al., 2011) framework. Using odMLtables (Sprenger et al., 2019) it is possible to organize and store complex metadata in a hierarchical format and collect, manipulate, visualize, and store metadata in tabular representations. However, this platform imposes no structure on individual files that are generated during experiments, which may lead to metadata e.g., not being stored alongside data in a modularized and searchable fashion and may thus hamper shareability and usability.

The above data management systems and tools either impose little structure on the stored data or metadata, leaving it up to the researcher to design a custom storage specification, or assume particular fields that need to be predefined such as in DataJoint^1^. However, research is dynamic in nature and new discoveries often change what data and metadata within datasets should be in focus. An ideal data management solution for neuroscience laboratories needs to be flexible and adaptable to various experimental paradigms (Denker and Grün, 2016).

Alyx^2^ is a notable exception that for the most part has few assumptions about the metadata to be stored, and allows its users to store arbitrary metadata in JSON fields. However, like many other data management solutions, Alyx requires manual installation, configuration, and maintenance of a server to be used in a multi-user environment. Solutions that instead are based on existing hosting providers can significantly lower the threshold for adapting a data management solution in a laboratory.

To address the shortcomings of existing solutions, we have created Expipe, a flexible, lightweight system for data handling. We propose a semi-structured data management platform that is lightweight in nature and requires little planning and maintenance to facilitate a broad range of experiments in neuroscience. Being modular and providing both human and machine readable metadata Expipe also support provenance tracking with GIN^3^ and Git Large File Storage^4^.

**Figure 1:**
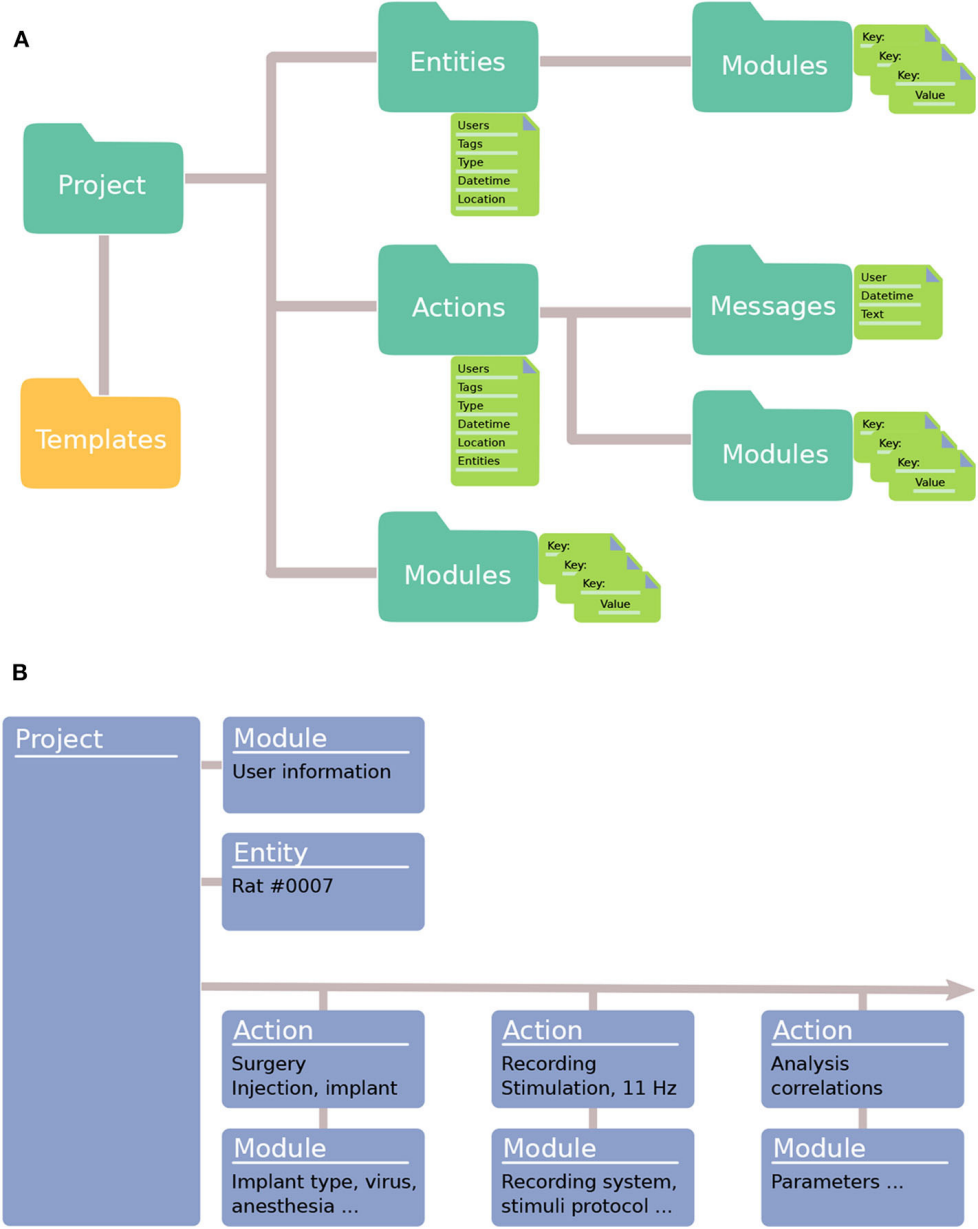
Expipe data model. **(A)** An Expipe Project contains Entities and Actions. Entities represent the long-lived elements in a project (e.g., experimental subjects). Actions define events that occurred at a certain time (e.g., an experiment or a surgery). Modules contain metadata about a Project, an Action, or an Entity. Templates can be used to pre-define Modules. **(B)** A typical working example, using Expipe to structure an experimental pipeline. In this example, there is one Entity (experimental subject: Rat #0007) and three Actions with correspondent Modules: a surgery, a recording, and an analysis.

To organize metadata for data collected in the lab, an Expipe Project contains the following objects: Modules, Actions, Entities, and Templates (Figure 1A). The concepts are abstract, making Expipe flexible to use in many different scenarios. Also, we made the concepts few and simple to avoid introducing an overly abstract framework that appears foreign to other researchers.

As dataset sizes can grow very quickly, making it slow to explore a scientific project, the capability of querying metadata alone is essential to get an overview of the project and possibly to select subsets of the database for further processing. In Expipe, **Modules** sit at the core of the system and contain metadata describing Projects, Actions and Entities in detail. The Modules typically specify metadata about the equipment, environment, or subjects, such as the numerical aperture of a microscope lens, the serial number of an acquisition system, or the temperature of a room. **Actions** define events that occurred at a specific time, such as an experiment, an analysis, or a simulation (Figure 1B). Actions have a few specific attributes, such as a timestamp, and store detailed metadata in Modules. **Entities** are long-lived things that are used in an Action, typically the ID of an experimental subject. Actions refer to Entities, but they do not link directly to them. Messages are user specific lines of text added to actions, such as notes. As Modules can be tedious to define each time an Action is created, **Templates** can be used to ease this process by holding predefined information typically added to Modules. We will cover Expipe objects in more detail in section 2.

A common obstacle in designing a general data management solution for experimental data is to choose the right database schema in advance. For that reason, Expipe uses a NoSQL key-value database model which is flexible in terms of defining the metadata to store. Rather than forcing the user to select a database schema ahead of time, Expipe uses implicit schemas in the form of what we call module Templates. These are similar in scope to odML terminologies and to a large extent also compatible with odML. Templates can be used to create Modules, which are a *snapshot* of the Template at the time of creation. Templates can change over time to reflect changes in the Project without affecting existing Modules, since the Modules are copied from, rather than linked to Templates. Records in a relational database, in comparison, are tied to a schema.

Expipe is portable and has few dependencies. By default, Expipe uses the file system for storing metadata, which means that no additional database installation or configuration is required. Moreover, we have written a reference implementation in Python and an extendable command line interface (CLI), making Expipe widely available to the scientific community. The Python API allows users to interact with Expipe programmatically. Additional Jupyter extensions are included with the API to provide a graphical user interface (GUI) that gives an overview of stored contents.

Expipe is written with modularity in mind and can use NoSQL databases as backends, such as a Google Firebase^5^. However, the filesystem is used as a backend by default. One benefit with the filesystem backend is that it allows data to be stored close to the metadata, within the Expipe directory structure. The filesystem backend also allows Expipe to easily be combined with GIN or Git LFS to get full version control, safe synchronization between collaborators, and hosting for data sharing.

Our goal has been to make Expipe a lightweight framework that can be adopted and used by researchers in laboratories with immediate data handling needs. To this end we present the Expipe data model and envisioned usage below.

## 2. Expipe Walk-Through

In this section we will present a step-by-step walk-through to the Expipe framework, by setting up an Expipe project for a sample application from neuroscience involving open-field foraging experiments on rats combining extracellular recordings of medial entorhinal cortex (MEC) and optogenetic stimulation. Expipe is available on PyPI^6^ and can be installed with pip. For documentation, we refer to https://expipe.readthedocs.io.

### 2.1. Project

First of all, we need to create an Expipe project. To create a Project using the Python API for Expipe, one simply needs to import the expipe package and run the create_project function:


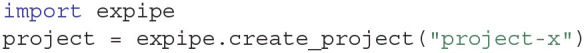


Expipe, by default, utilize the filesystem as a backend. This means that an organized set of folders and files are used. When our “project-x” is created, an Expipe folder named project-x will be created in the current working directory.

An Expipe project will contain a collection of Actions, Entities, Templates, Messages and project Modules (Figure 1A), which we will explain in the following sections. A typical working example of how to structure an experimental pipeline with Expipe is illustrated in Figure 1B.

### 2.2. Entities

Entities represent physical or conceptual things, such as experimental equipment or subjects (like rats and mice). In our simple example, we assume we are using a single rat (ID 0007) for our experiments. We can then create the “rat” Entity using the Project.create_entity function:


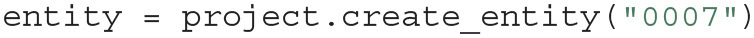


Similarly to the project creation, the above command will create a folder “0007” in the Entity folder of the project. All Entities have some common attributes, such as tags, users, location, type and datetime, which can be easily accessed and modified as follows:


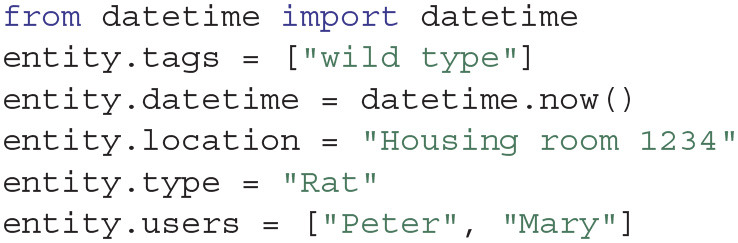


Entities are not static, but they can be updated over time following the course of a project. In our example, for instance, the Entity will undergo a modification when a surgery is performed, when a recording is made, or when the animal is euthanized. These types of modifications can further be described with Expipe Actions.

### 2.3. Actions

Actions represent things that have happened at a specific point in time, such as an experiment, an analysis, a surgery, or a simulation run. In our toy project, after we have performed an experiment, we can register it as an Action using the Project.create_action function:


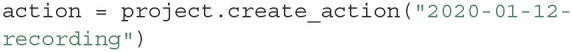


Actions can be updated over time (for instance, by adding processed data after some analysis). All Actions have some common attributes, such as tags, users, location, type, entities and datetime. In our example, we performed a 11 Hz optogenetics stimulation during the recording, hence we can add this piece of information as tags:


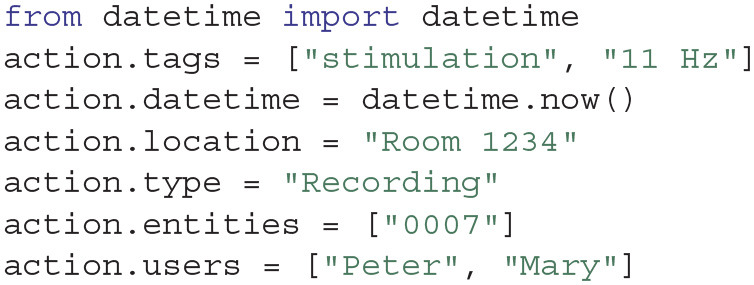


These attributes are stored in the attributes.yaml text file in the Action folder.

### 2.4. Modules

So far, we have only handled common metadata for Actions and Entities, such as tags, dates, and users. However, further specific metadata can be stored using Modules. Actions, Entities and the Project as a whole can have Modules attached. A Module holds metadata in key-value form, which is similar to a map or hash table in popular programming languages. Modules are intended to hold metadata such as the equipment in an experiment, the protocol that was used, or a summary of the obtained results. For example, a Module could describe the arena for the open-field experiment that we performed as follows:


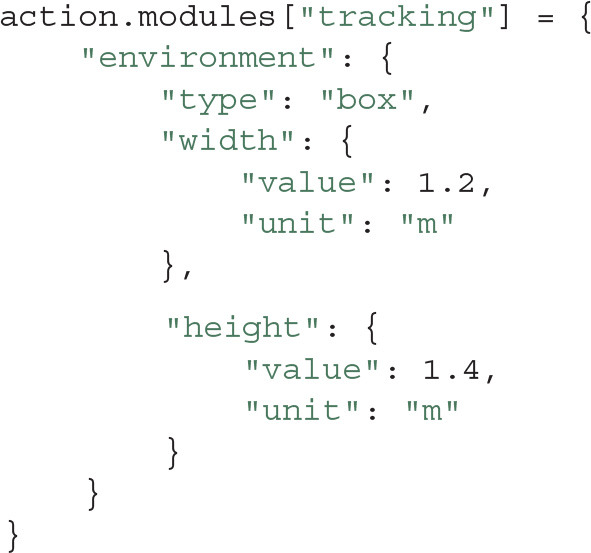


Within the Action's folder there is another folder called modules, which contains each Module as a YAML file. The above code snippet would for instance produce a file name tracking.yaml with the following contents:


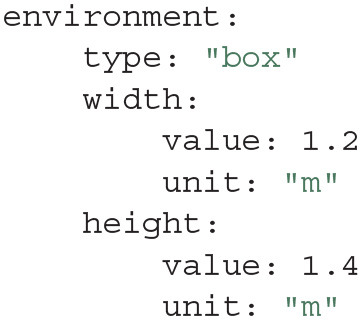


This means that the metadata can be easily modified later, not only using the Python API, but also by manually editing the file in a text editor. Note that when using Expipe in combination with provenance tracking systems such as Git or GIN, these types of changes will be documented and thus not pose a risk for corruption of metadata integrity. The simple YAML syntax makes editing easy, without the need for a separate GUI only for editing purposes. Since many Actions could share the same metadata (e.g., several recording using the same open-field arena), the creation of Modules is facilitated by Templates.

### 2.5. Templates

Modules can be created from scratch, as above, or automatically be included based on a Template, by passing the template argument:


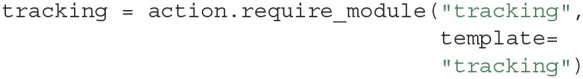


This will copy the entire Template named tracking into a Module with the same name in the given Action. As some metadata can be Action-specific, the Module can then be edited, for instance by filling out any blank values in the Template, either manually or by using the Python API:


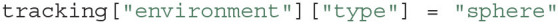


In addition to Action Modules, Templates can also be used to instantiate Modules for Entities and the entire Project (Figure 1A).

There is minimal linking between metadata in Expipe to improve provenance. In a relational database, an Action would typically have links to the equipment used in a many-to-many relationship. However, Expipe is instead designed to copy the entire equipment Template into the Modules of the Action. This is to ensure that the exact state of the equipment is recorded in the Action, and removes the risk of inadvertently updating the state of the equipment for an existing Action.

### 2.6. Messages

When performing an experiment, it is important for the experimenter to log some messages as a future reminder for the analysis, such as noting that a recording channel is noisy or that possibly a good unit is found. In order to keep a virtual laboratory book, Messages can be added to an Action to add notes and comments:


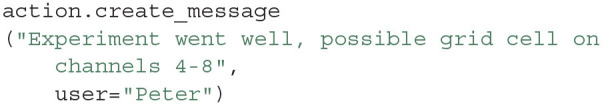


Messages are given a timestamp (the time of creation if not otherwise given), and stored within the Action.

### 2.7. Data

Actions, such as recordings, are usually performed by acquiring experimental data. Data can be easily linked to an Action in Expipe by using the data property of an Action, which is a map from a string ID (e.g., “tracking”) to a path relative to the data folder of an Action:


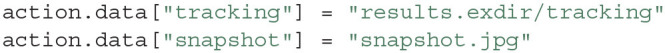


Here, exdir (Dragly et al., 2018) is used as the storage format.

The absolute path of the file is retrieved as a native pathlib path by calling the Action.data_path function:


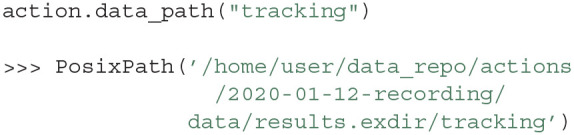


By default the path is assumed to be stored relative to the action and the absolute path can be obtained with Action.data_path. However, it is possible to use the data field to store any string, for example, pointing to a directory on a server:


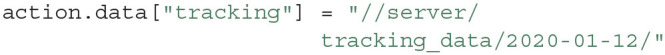


We recommend storing the data directly in the “data” folder of an Action, since the data and metadata can be tracked together by version control systems such as GIN, or Git LFS.

### 2.8. Expipe Command Line Interface

The command line interface (CLI) provides minimal interaction with the Expipe environment. The CLI can be used to create and configure projects, and to list available Actions, Entities and Templates.

It is easily extendable to add user specific functionalities by making an Expipe plugin. The addition of a plugin is performed in two stages. First, a Python package (named my_package in the following example) must be installed in the Python environment. Then, using the click Python package^7^, one can create a subclass of the expipecli.utils.plugin.IPlugin class and define the required commands within the attach_to_cli method:


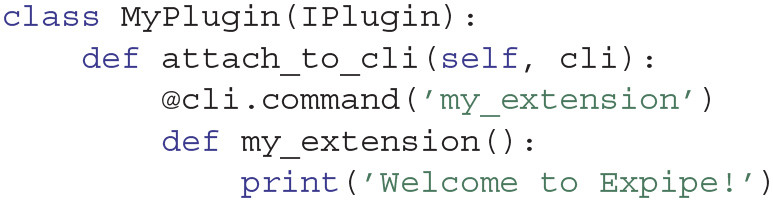


Finally, the newly created plugin must be added to the expipe Expipe framework:


expipe config global --add plugin my_package


The newly created CLI command can be now invoked through the Expipe CLI:


>>> expipe my_extension

Welcome to Expipe!


For a comprehensive plugin used by the CINPLA laboratory to register, store, and analyze experimental recordings, we refer to the expipe_plugin_cinpla package (https://github.com/CINPLA/expipe-plugin-cinpla).

### 2.9. Exploring Expipe Projects

When an Expipe project has been created and populated, it can be explored through the API by simply looking in the filesystem (if this is the preferred backend) or with a Graphical User Interface (GUI).

**Figure 2:**
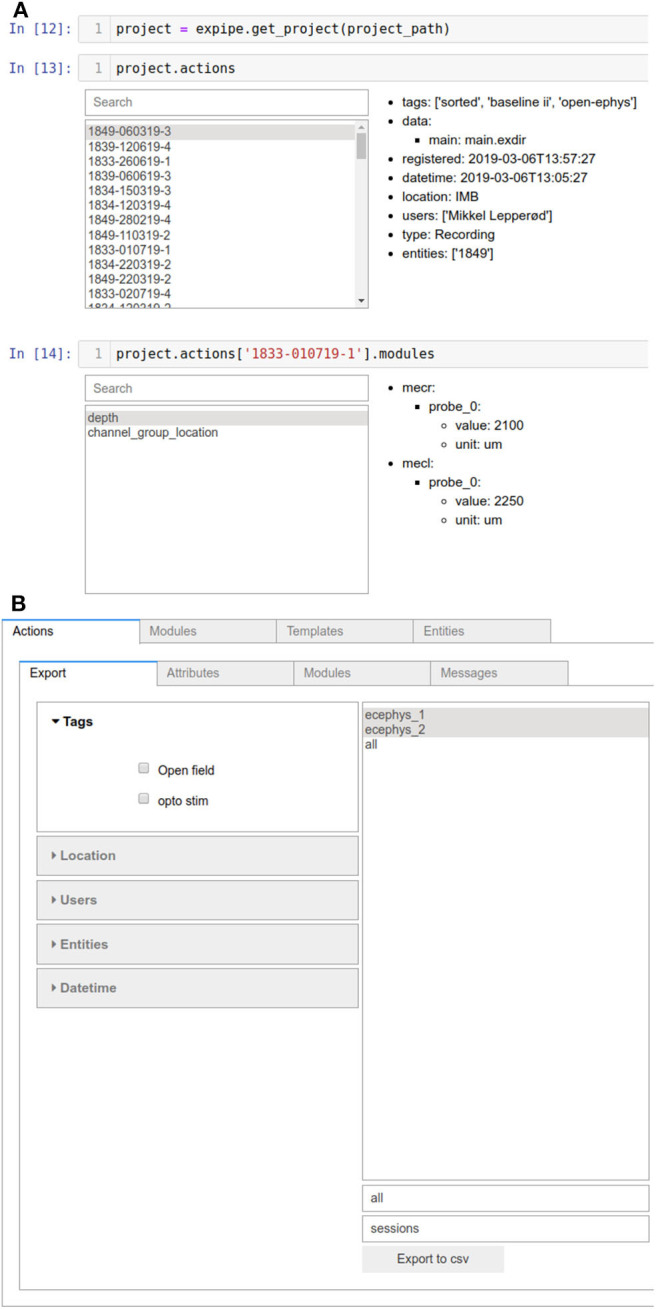
Graphical User Interface with Ipython widgets. **(A)** Simple overview GUI obtained when an Expipe object container (such as project.actions) is run in a Jupyter cell. **(B)** For a more comprehensive overview the Browser can also be attained, where the entire Project structure is indexed.

A basic GUI is available when using Expipe in a Jupyter notebook^8^. This GUI is based on IPython Widgets^9^. The widgets can be spawned by simply running the Expipe objects in a Jupyter cell, as shown in Figure 2. In addition, an entire Expipe Project can be visualized using the available Browser:


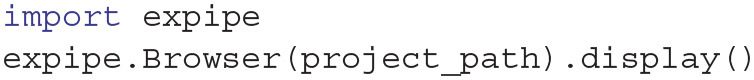


In the Browser GUI all Actions with their attributes are indexed enabling the user to get an overview of the entire Project structure, and contents such as attributes, Messages, and Modules.

Expipe objects support queries like searching for Actions, Entities, and Modules, either through the GUI or with custom scripts by means of attributes. To perform more complex queries it can be convenient to combine object attributes such as tags etc. and metadata into a structured database, e.g., using Pandas^10^. The Expipe Browser allows to export all Actions and attributes to comma-separated values (CSV) file, which can then be e.g., loaded in Pandas. To include information from modules, custom scripts must be written, or if modules are created with odML, these can be combined with odMLtables (Sprenger et al., 2019).

Through the Python API, Expipe objects can be conveniently accessed as dictionaries in order to ease iteration, retrieval and setting. Actions, Modules and Entities can be iterated directly using values(), for both key and values items() is preferred


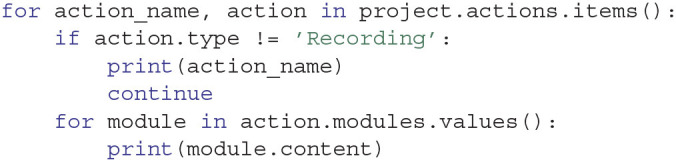


## 3. Discussion

In contemporary neuroscience, innovation happens also with discoveries of new types of measurement techniques and data. These may differ profoundly from existing data, when for example a new behavioral acquisition is added to electrophysiology or imaging setup. Such changes in the collected data within a project require a flexible data management system.

In this paper, we present Expipe—a data management solution for neuroscience laboratories. Differently from existing solutions (HarkȩŻlak et al., 2014; Olsson and Hartley, 2019; Sprenger et al., 2019)^1^, Expipe provides a semi-structured, but flexible data management solution specifically designed to encompass the life-cycle of experimental projects in neuroscience. Being lightweight and simple, relying on the familiar file-system backend to organize project components, we see Expipe as an accessible and easy-to-use tool for laboratories to start implementing a reproducible data management system in their research. This is a first and important step for (many) research groups that only use an *ad-hoc* solutions for organization of data and metadata.

The Expipe structure based on Entities, Actions, and Modules shares similarities with the core structures of PROV-DM^11^ (Entity, Activity, and Agent). The descriptions of an Entity is very similar in scope of what we envisioned as Entities. Activities are similar to Actions, although there is no required link between an Action and an Entity in Expipe. Finally, instead of an Agent, we have chosen to optionally have a user to be specified with an Action or an Entity.

One of the main strengths of Expipe is its flexibility. However, flexibility can also be considered as a limitation. The definition of metadata (Modules) is left to the user, but we encourage the use of predefined Templates for data collection, ideally standardized by the scientific community, e.g., using odML terminologies (https://terminologies.g-node.org/v1.1/terminologies.xml). Odml, however, is not designed as a database, rather as a way to structure metadata, one experiment at a time. Expipe can work together with odML to give structure and modularization, with Expipe providing structure to the Project (e.g., each experiment is an Action belonging to the project) and with odML giving structure at the metadata level, by using well-defined and community-accepted metadata fields.

Another possible limitation of the Expipe framework is related to provenance. Our relaxed integrity verification in relations between objects simplifies structure and development, but also comes with some drawbacks. For instance, when adding an Entity to an Action, there is no insurance that this Entity exists or is described. Similarly, if a user is added to an Action attribute, the user name might, for instance, be incorrectly spelled. The structure in Expipe thus relies on its users to ensure provenance. Methods for user specific schemas that ensure provenance could be added through plugin functionality, e.g., by building a stricter control of object creation and annotation. For example, the click Python package required to create custom Expipe plugins provides a first check on argument types. Finally, an extended plugin functionality that accepts schemas at project creation could be added, this would also ease integration of Expipe into more complete data handling solutions.

A typical project in neuroscience may contain many experiments, but only a subset of the experiments might be selected for further analysis. In this situation it is highly convenient to be able to efficiently search for indicators that signify inclusion in such a subset. This kind of search can be done by using Action attributes such as tags.

Because of the lightweight nature of Expipe, it can easily be integrated with other data management software. For instance, workflows written in Snakemake (Köster and Rahmann, 2012) can depend on files in an Expipe structure to define an automated analysis workflow. Data sharing platforms that are based on the file system, such as GIN, can easily track the files in an Expipe folder. Other tools, such as Git and Git LFS or Perforce^12^ for version control, can also be used in combination with Expipe, with Git LFS being the preferred solution in our lab.

Expipe does not impose any restriction on file formats, to improve flexibility and to enable dealing with different types of data. In our lab, we have used the Exdir format (Dragly et al., 2018), which we developed as an alternative to HDF5, together with Expipe in several projects. Alternatively, a common standard that is being increasingly used by the neuroscience community and that we strongly recommend is Neurodata Without Boarders (NWB) (Teeters et al., 2015). Other common file formats, such as image sequences, HDF5, and video files can be stored in the data directory of any Action. There is no limitation in Expipe to the types of files it can point to.

Finally, Expipe uses the file system as backend for projects. However, this is not the only available solution. A Firebase backend is also supported, which stores the entire project as key value pairs using Google Firebase^5^. The file system backend could also support integration to cloud-based systems, such as Dropbox^13^, Google^14^, or Amazon S3^15^.

## 4. Conclusion

Experimental progress in neuroscience is often innovative in terms of how behavioral and data acquisition paradigms are used and combined. In such cases it can be difficult to *a priori* design a data and metadata structure that encompass all aspects of a project. On the other hand, having no structure at all can lead to problems with reproducibility and sharability. To solve this problem we propose a semi-structured data management platform that is lightweight in nature and requires little planning and maintenance to facilitate a broad range of experiments in neuroscience. Being modular and providing both human and machine readable metadata in text files Expipe can easily be combined with other tools such as odMLtables, Pandas, Git and Git LFS. Moreover, it is easy to search and create subsets of experiments within a large project making Expipe ideal both during data acquisition and data analysis. Expipe is a novel data management tool that solves many of the problems associated with existing data and metadata management software. Our hope is that Expipe will be adopted by the community and become a simple data management solution that can be integrated with other software for analysis workflows, provenance tracking, and data sharing.

With Expipe we propose that a modularized semi-structured database model can enable an efficient and user friendly approach to handling complex experimental datasets.

## 5. Significance Statement

To facilitate data sharing, provenance and management of data and metadata we introduce Expipe, a semi-structured and lightweight data management platform designed for neuroscience research. Expipe implements a conceptually simple and familiar project structure and includes functionalities for data and metadata handling, retrieval, and exploration, which in turn can simplify the steps from experiments to analysis. Differently from existing solutions, the flexible and easy-to-use Expipe framework can provide an entry-level data management solution for both small and large experimental laboratories.

## Data Availability Statement

The source code of Expipe is available at github.com/cinpla/expipe.

## Author Contributions

ML conceived the project, wrote the code, and wrote the manuscript. S-AD conceived the project, wrote the code, and wrote the manuscript. AB and MM wrote the code and wrote the manuscript. AM-S acquired funding. TH and MF acquired funding and wrote the manuscript. All authors contributed to the article and approved the submitted version.

## Conflict of Interest

The authors declare that the research was conducted in the absence of any commercial or financial relationships that could be construed as a potential conflict of interest.

## Footnotes

^1^https://datajoint.io/

^2^alyx.readthedocs.io

^3^https://gin.g-node.org/

^4^git-lfs.github.com

^5^firebase.google.com

^6^https://pypi.org/project/expipe/

^7^https://click.palletsprojects.com/

^8^https://jupyter.org/

^9^https://ipywidgets.readthedocs.io/

^10^https://pandas.pydata.org/

^11^https://www.w3.org/TR/prov-dm/

^12^perforce.com/products/helix-core

^13^https://www.dropbox.com/

^14^console.cloud.google.com/

^15^aws.amazon.com/s3
